# Machine learning for predicting breast-conserving surgery candidates after neoadjuvant chemotherapy based on DCE-MRI

**DOI:** 10.3389/fonc.2023.1174843

**Published:** 2023-08-09

**Authors:** Zhigeng Chen, Manxia Huang, Jianbo Lyu, Xin Qi, Fengtai He, Xiang Li

**Affiliations:** Department of Radiology, the Second Hospital of Dalian Medical University, Dalian, China

**Keywords:** machine learning, breast cancer, neoadjuvant chemotherapy, magnetic resonance imaging, breast-conserving surgery

## Abstract

**Purpose:**

This study aimed to investigate a machine learning method for predicting breast-conserving surgery (BCS) candidates, from patients who received neoadjuvant chemotherapy (NAC) by using dynamic contrast-enhanced magnetic resonance imaging (DCE-MRI) obtained before and after NAC.

**Materials and methods:**

This retrospective study included 75 patients who underwent NAC and breast surgery. First, 3,390 features were comprehensively extracted from pre- and post-NAC DCE-MRIs. Then patients were then divided into two groups: type 1, patients with pathologic complete response (pCR) and single lesion shrinkage; type 2, major residual lesion with satellite foci, multifocal residual, stable disease (SD), and progressive disease (PD). The logistic regression (LR) was used to build prediction models to identify the two groups. Prediction performance was assessed using the area under the curve (AUC), accuracy, sensitivity, and specificity.

**Results:**

Radiomics features were significantly related to breast cancer shrinkage after NAC. The combination model achieved an AUC of 0.82, and the pre-NAC model was 0.64, the post-NAC model was 0.70, and the pre-post-NAC model was 0.80. In the combination model, 15 features, including nine wavelet-based features, four Laplacian-of-Gauss (LoG) features, and two original features, were filtered. Among these selected were four features from pre-NAC DCE-MRI, six were from post-NAC DCE-MRI, and five were from pre-post-NAC features.

**Conclusion:**

The model combined with pre- and post-NAC DCE-MRI can effectively predict candidates to undergo BCS and provide AI-based decision support for clinicians with ensured safety. High-order (LoG- and wavelet-based) features play an important role in our machine learning model. The features from pre-post-NAC DCE-MRI had better predictive performance.

## 1 Introduction

Breast cancer is the most prevalent cancer among women and its incidence is increasing yearly worldwide (1). Neoadjuvant chemotherapy (NAC) is the standard treatment for early breast cancer (2). For patients with heavy tumor load, it is designed to reduce tumor stage and surgical interventions, provide more patients with opportunities for breast-conserving surgery (BCS), and avoid axillary lymph node dissection (3–5). For human epidermal growth factor receptor 2 (HER2) + or triple-negative (TN) breast cancer, NAC can provide doctors with more *in vivo* information regarding drug sensitivity, a so-called individual drug-sensitive platform (6). With the development of new targeted drugs for early breast cancer, both the population receiving NAC and the rate of achieving pathological complete response (pCR) are increasing (7, 8). Meanwhile, patients without pCR still have the opportunity to downstage to BCS through NAC, which can cause less damage to the breast (9, 10). Thus, safe selection of candidates for BCS after NAC is a critical issue.

The pCR rate varies among breast cancer subtypes, and HER2+ status is more likely to result in pCR (11). However, evaluation of pCR is not sufficient to identify candidates for BCS. The efficacy response of breast cancer patients after NAC can be classified into three categories: pCR, partial remission, and non-remission. Partial remission can be further divided into single-lesion shrinkage, major residual lesions with satellite foci, and multifocal residuals based on microscopic morphology (12–14). Fukada et al. (15) classified tumors into concentric shrinkage (CS) and non-CS patterns. The CS pattern was associated with better disease-free and overall survival rates. However, in their study, the CS was composed of single lesion shrinkage and major residual lesions with satellite foci. Wang et al. (16) further specified this by defining single lesion shrinkage as type 1, multifocal and patchlike lesions as type 2, and major residual lesions with satellite foci as type 3. They proposed that types 2 and 3 in partial remission had relatively high recurrence rates after undergoing BCS. This is because types 2 and 3 have the risk of missing tiny lesions, and negative surgical margins are not guaranteed. Therefore, a detailed differentiation of tumor shrinkage patterns is necessary for clinical work. The European Society of Breast Imaging (EUSOBI) recommends magnetic resonance imaging (MRI) to evaluate the efficacy of NAC in breast cancer (17). Dynamic contrast-enhanced MRI (DCE-MRI) is a technique for contrast imaging that uses differences in the distribution of contrast agents within the tissues. It serves as the most sensitive MRI sequence for breast cancer, allowing simultaneous assessment of tissue perfusion and morphological changes to reflect the response of breast tumors to NAC (18). Machine learning (ML) can be used to extract information that cannot be recognized by clinicians in medical images (19). Thus, ML can greatly improve the ability to evaluate NAC with MRI, and most studies have focused on building an ML model to distinguish pCR from non-pCR (20). Previous studies have reported that multiparametric MRI performed better than single sequences for prediction (21, 22). Nevertheless, in multiparametric MRI radiomics, the outlining of the region of interest (ROI) is usually performed in DCE-MRI and applied to other sequences using image registration algorithms (23). The Speeded Up Robust Features algorithm, an accelerated version of the scale-invariant feature transform algorithm, still suffers from problems, such as insufficient feature points and accuracy (24). Another way to outline the ROI is to complete the ROI on all sequences separately (25), but the shortcoming is that the differences in the ROI of each sequence are difficult to avoid. In addition, general radiomics features, such as texture features, and first-order features, have been adequately analyzed, while the use of high-order features is relatively inadequate.

The image before the first phase of NAC is called the baseline image. The image obtained after the last phase of NAC and before surgery is referred to as the preoperative image. We used pre-NAC DCE-MRI as the baseline image and post-NAC DCE-MRI as the preoperative image. In this study, we constructed a model that has the potential to provide clinicians with appropriate candidates for BCS based on pre- and post-NAC DCE-MRIs.

## 2 Materials and methods

### 2.1 Patients

This study was approved by the Institutional Review Board, and the requirement for informed consent was waived. This study included 75 patients with breast cancer at the Second Hospital of Dalian Medical University between June 2014 and May 2021. Pre- and post-NAC images of the patients were used for the analysis, and the total number was 150. The inclusion criteria were as follows (1): invasive breast cancer confirmed by biopsy (2), accepted MRI examinations before and after NAC (3), underwent definitive surgery after standard NAC in our hospital, and (4) available pathologic results. The exclusion criteria were as follows (1): receiving other treatments during NAC. The ratio of the training set to validation sets was 4:1.

The chemotherapy regimen for all enrolled patients was based on the standard regimen recommended by the National Comprehensive Cancer Network (NCCN) Breast Cancer Guidelines. The preoperative chemotherapy regimens were taxane-based, anthracycline-based, or a combination of both. For HER2+ patients, the addition of anti-HER2 therapy (e.g., trastuzumab or a combination of trastuzumab and pertuzumab) is required.

### 2.2 MRI technique and immunohistochemistry

All patients were examined with 1.5T or 3.0T breast MRI (GE Signa HDxt 1.5T, GE Discovery MR 750 W 3.0T, Siemens Verio 3.0T) before and after NAC (**Table 1**). Axial DCE-MRI: A T1-weighted pre-contrast scan was first performed, followed by injection of a contrast agent (Gd-DTPA). After injection, 20 ml of saline was used to flush the tube, which was then continuously scanned for nine phases.

**Table 1:** DCE-MRI protocol for each scanner.

DCE-MRI	GE Signa HDxt 1.5T	GE Discovery MR 750W 3.0T	Siemens Verio 3.0T
TR (ms)	5.1	6.9	4.54
TE (ms)	2.5–12	minimum	1.61
Matrix (pixels)	320 × 384	288 × 320	346 × 384
FOV (mm)	320 × 320	360 × 360	340 × 340
Slice thickness (mm)	2.8	1.4	1.6
Flip angel	15	10	10

TR, repetition time; TE, echo time; FOV, field of view.

### 2.3 ROI masking

Two radiologists assessed the tumor borders. A radiologist with 5 years of experience completed ROI masking and was then reviewed by another radiologist with over 23 years of experience. The two radiologists reached a consensus on tumor bounderies. The tumor contours on each slice of the third post-enhanced image were manually outlined, and a 3D volume of interest (VOI). This step was performed on pre-NAC images and post-NAC images separately by using 3D Slicer 4.10.2 (www.slicer.org, including the following steps unless noted).

### 2.4 Pathological assessment

Biopsies and surgical specimens were handled by a pathologist with more than 8 years of experience. Surgical specimens were fixed in standard formalin solution and processed in a standard breast tissue processor, according to which the longest diameter of the tumor was recorded.

Immunohistochemistry was used to determine the expression of Ki-67, progesterone receptor (PR), estrogen receptor (ER), and HER2. HER2 expression was graded as 0, 1+ was negative, and 3+ was positive. If HER2 expression was graded as 2+, additional fluorescence *in situ* hybridization was required.

The maximum diameter of the tumor was measured using a 3D slicer in pre-NAC DCE-MRI as the baseline. Shrinkage patterns were assessed by comparing the surgical specimens with the baseline values. The definition of shrinkage patterns was based on the Response Evaluation Criteria in Solid Tumors (RECIST) version 1.1, Chalian et al., Schwartz et al. (26–28). Type 1 shrinkage pattern included pCR and single-lesion shrinkage. pCR was defined as no residual invasive carcinoma in the primary lesion or axillary lymph node after NAC, and single lesion shrinkage was defined as a lesion that had shrunk by more than 30% of its longest diameter. Type 2 shrinkage patterns include major residual lesions with satellite foci, multifocal residuals, stable disease (SD), and progressive disease (PD). Major residual lesions with satellite foci were defined as the main residual lesions accompanied by at least one minor lesion on the continuous slides. Multifocal residuals were defined as the presence of at least two separate lesions. SD was defined as a lesion shrinking by less than 30% of its longest diameter, and PD was defined as a lesion exceeding the baseline in its longest diameter. Two experienced pathologists independently examined the specimens and agreed to the regression patterns.

### 2.5 Image processing and features extraction

Non-uniform intensity normalization (N4) bias correction was applied to extract the bias field in MR imaging and correct it to eliminate the effect of artifacts. The voxel sizes of the images were resampled to (1, 1, 1).

The features of the pre-NAC model (pre-NAC features) were extracted from pre-NAC DCE-MRI, and those of the features of post-NAC model (post-NAC features) were extracted from post-NAC DCE-MRI. The features of the pre-post-NAC model (pre-post-NAC features) were obtained by subtracting the value of the post-NAC features from the pre-NAC features. The features of the combination model (combination features) consisted of pre-NAC, post-NAC, and pre-post-NAC features.

### 2.6 Statistical analyses

All features were imported into the Darwin Scientific Research Platform (Medical AI Technology (Beijing) Co., Ltd.) for subsequent operations. The feature values were normalized between −1 and 1 by maximum absolute value normalization. Minimum redundancy maximum relevance (mRMR) feature selection was utilized to select highly relevant features from pre-NAC, post-NAC, pre-post-NAC, and combination features. The filtered features were used to construct logistic regression (LR) models. LR is a model for solving binary classification problems. The importance of each independent variable is quantified in LR, and a set of independent variables with optimal classification performance is utilized to form a linear combination (29). As an optimization problem, the binary class L2 penalized LR minimizes the following cost function:


$$y=\underset{w,c}{\text{min }}\frac{1}{2}w^{⊤}w+C\sum_{i=1}^{n}\text{log}\left(\text{exp}\left(-y_{i}\left(x_{i}^{⊤}w+c\right)\right)+1\right)$$

$x_{i}$ is the radiomics features for sample i , $y_{i}$ is the sample i label, w is the coefficient vector of the LR model, and c the inverse of the regularization intensity.

The performance of the LR model was demonstrated by the receiver operating characteristic curve. Then the area under the curve (AUC), accuracy, sensitivity, and specificity were calculated. Type 1 was defined as negative and type 2 was defined as positive.

The patient characteristics were calculated by SPSS software (version 26, IBM). The normally distributed continuous data were expressed as mean ± standard deviation and examined by independent t-tests. Continuous variables between two groups were examined by Fisher’s exact test or chi-square test. 0.05 was used as the significant level.

## 3 Result

### 3.1 Patient characteristics

**Figure 1:**
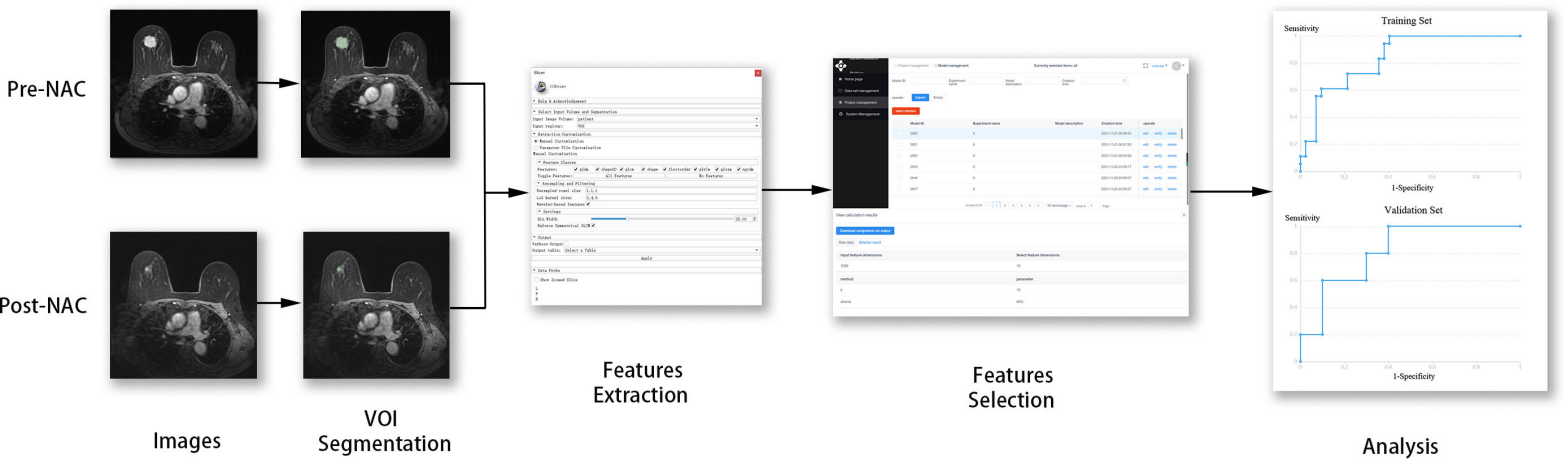
Radiomics workflow.

The radiomics framework is shown in **Figure 1**. A total of 75 patients were enrolled in the study, in which type 1 accounted for 67.2% (53.2 ± 9.1 years), and type 2 for 32.8% (53.3 ± 8.8 years). Premenopausal patients accounted for 48.1% and 47.8% of patients with type 1 and type 2 diseases, respectively. ER >1% accounted for 61.5% of type 1 cases and 60.9% of type 2 cases. A PR >1% accounted for 57.7% of type 1 cases and 52.2% of type 2 cases. HER2 positive accounted for 57.7% of the type 1 cases and 65.2% of the type 2 cases. Clinical T-stages 1–2 accounted for 78.8% and 65.2% of patients with type 1 and type 2 tumors, respectively. Luminal A, luminal B, HER2, and TN accounted for 13.5%, 59.6%, 17.3%, and 9.6% in type 1 while 21.7%, 39.1%, 34.8%, and 4.3% in type 2. Age (*P* = 0.947), menopausal status (*P* = 0.984), ER status (*P* = 0.956), PR status (*P* = 0.657), clinical T-stage (*P* = 0.211), and molecular subtype (*P* = 0.215) were not significantly different between types 1 and 2. The expression level of Ki-67 was significally different (*P* = 0.047) between type 1 and type 2, of which the proportion of Ki-67 >20% in type 1 was higher (78.8% in type 1, 56.5% in type 2). The characteristics of the type 1 and type 2 patients are shown in **Table 2**. There were no significant differences in the patient characteristics between the training and validation sets (**Table 3**).

**Table 2:** Characteristics of patients and breast cancers in our study.

Characteristics	Type 1	Type 2	*P*-value
No. of patients	52	23	
Age, years	53.2 ( ± 9.1)	53.3 ( ± 8.9)	0.947
Menopausal Status			0.984
Premenopausal	25 (48.1%)	11 (47.8%)	
Postmenopausal	27 (51.9%)	12 (52.2%)	
ER Status			0.956
≤1%	20 (38.5%)	9 (39.1%)	
>1%	32 (61.5%)	14 (60.9%)	
PR Status			0.657
≤1%	22 (42.3%)	11 (47.8%)	
>1%	30 (57.7%)	12 (52.2%)	
HER2 Status			0.540
Positive	30 (57.7%)	15 (65.2%)	
Negative	22 (42.3%)	8 (34.8%)	
Ki-67 Status			0.047
≤20%	11 (21.2%)	10 (43.5%)	
>20%	41 (78.8%)	13 (56.5%)	
Clinical T-stage			0.211
1–2	41 (78.8%)	15 (65.2%)	
3–4	11 (21.2%)	8 (34.8%)	
Molecular Subtype			0.215
Luminal A	7 (13.5%)	5 (21.7%)	
Luminal B	31 (59.6%)	9 (39.1%)	
HER2	9 (17.3%)	8 (34.8%)	
TN	5 (9.6%)	1 (4.3%)	

ER, estrogen receptor; PR, progesterone receptor; HER2, human epidermal growth factor receptor 2; TN, triple-negative.

**Table 3:** Characteristics of patients in training set and validation set.

Characteristics	Training set	Validation set	*P*-value
No. of patients	60	15	
Age, years	53.1 ( ± 9.3)	53.7 ( ± 7.6)	0.824
Regression pattern			1.000
Type 1	42 (70.0%)	10 (66.7%)	
Type 2	18 (30.0%)	5 (33.3%)	
Menopausal Status			0.131
Premenopausal	29 (48.3%)	4 (26.7%)	
Postmenopausal	31 (51.7%)	11 (73.3%)	
ER Status			0.101
≤1%	22 (36.7%)	9 (60.0%)	
>1%	38 (63.3%)	6 (40.0%)	
PR Status			0.816
≤1%	26 (43.3%)	7 (46.7%)	
>1%	34 (56.7%)	8 (53.3%)	
HER2 Status			0.556
Positive	37 (61.7%)	8 (53.3%)	
Negative	23 (38.3%)	7 (46.7%)	
Ki-67 Status			0.486
≤20%	16 (21.2%)	6 (43.5%)	
>20%	44 (78.8%)	9 (56.5%)	
Clinical T-stage			0.842
1-2	44 (73.3%)	12 (80.0%)	
3-4	16 (26.7%)	3 (20.0%)	
Molecular Subtype			0.425
Luminal A	8 (13.3%)	4 (26.7%)	
Luminal B	33 (55.0%)	7 (46.7%)	
HER2	13 (21.7%)	4 (26.7%)	
TN	6 (10.0%)	0 (0%)	

ER, estrogen receptor; PR, progesterone receptor; HER2, human epidermal growth factor receptor 2; TN, triple-negative.

### 3.2 Model effectiveness

**Figure 2:**
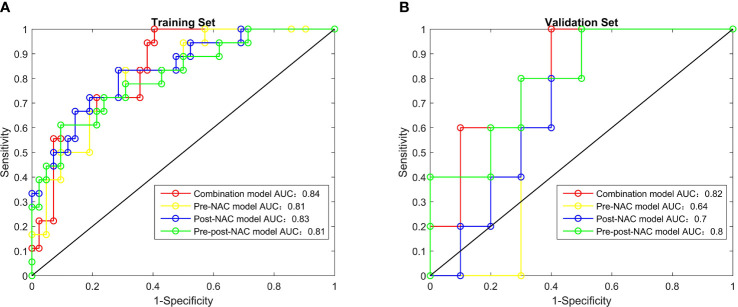
The receiver operating characteristic curves of radiomics features, combination model, pre-NAC model, post-NAC model and pre-post-NAC model in both the training set and the validation set, **(A)** is Training Set, **(B)** is Validation Set.

The AUC of combination model was 0.84 (95% CI: 0.74–0.94) for the training set and 0.82 (95% CI: 0.60–1.00) for the validation set, pre-NAC model was 0.81 (95% CI: 0.70–0.92) and 0.64 (95% CI: 0.34–0.94), post-NAC model was 0.83 (95% CI: 0.72–0.95) and 0.70 (95% CI: 0.43–0.97), pre-post-NAC model was 0.81 (95% CI: 0.69–0.93), and 0.80 (95% CI: 0.56–1.00) (**Figure 2**). The model performance details are listed in **Table 4**.

**Table 4:** Performance of different radiomics models in training and validation set.

	Training set				Validation set			
	AUC	ACC	SEN	SPE	AUC	ACC	SEN	SPE
combination model	0.84	0.75	0.72	0.76	0.82	0.73	0.80	0.70
pre-NAC model	0.81	0.72	0.83	0.67	0.64	0.60	0.80	0.50
post-NAC model	0.83	0.75	0.83	0.71	0.70	0.60	0.80	0.50
pre-post-NAC model	0.81	0.73	0.72	0.74	0.80	0.73	0.80	0.70

AUC, area under the curve; ACC, accuracy; SEN, sensitivity; SPE, specificity; NAC, neoadjuvant chemotherapy.

### 3.3 VOI features and linear combination

A total of 1,130 features were extracted from each VOI on pre- and post-NAC DCE-MRIs. The categories of features consisted of first-order features, shape features (2D and 3D), textural features, wavelet-based features, and 3D textural features from image data filtered by Laplacian-of-Gauss (LoG) with kernel sizes of 2, 4, and 6. After the feature selection, 15 features were filtered out. The selected features, in combination, are as follows:

pre-wavelet-HLH-gldm-Dependence Variance
pre-wavelet-HLL-glrlm-Short Run Low Gray Level Emphasis
post-original-shape-Elongation
pre-post-log-sigma-4-0-mm-3D-glcm-Informational Measure of Correlation
pre-post-log-sigma-4-0-mm-3D-glcm-Maximum Probability
pre-post-wavelet-HLL-glrlm-Run Entropy
post-original-shape-Maximum 3D Diameter
post-wavelet-HHL-glrlm-Short Run High Gray Level Emphasis
pre-post-log-sigma-6-0-mm-3D-gldm-Small Dependence Low Gray Level Emphasis
pre-wavelet-HLL-first order-Skewness
post-log-sigma-6-0-mm-3D-glcm-Maximal Correlation Coefficient
post-wavelet-HHH-gldm-Dependence Variance
pre-wavelet-LHH-gldm-Dependence Variance
pre-post-wavelet-HHH-glszm-Size Zone Non Uniformity Normalized
post-wavelet-LHL-gldm-Gray Level Non Uniformity

**Figure 3:**
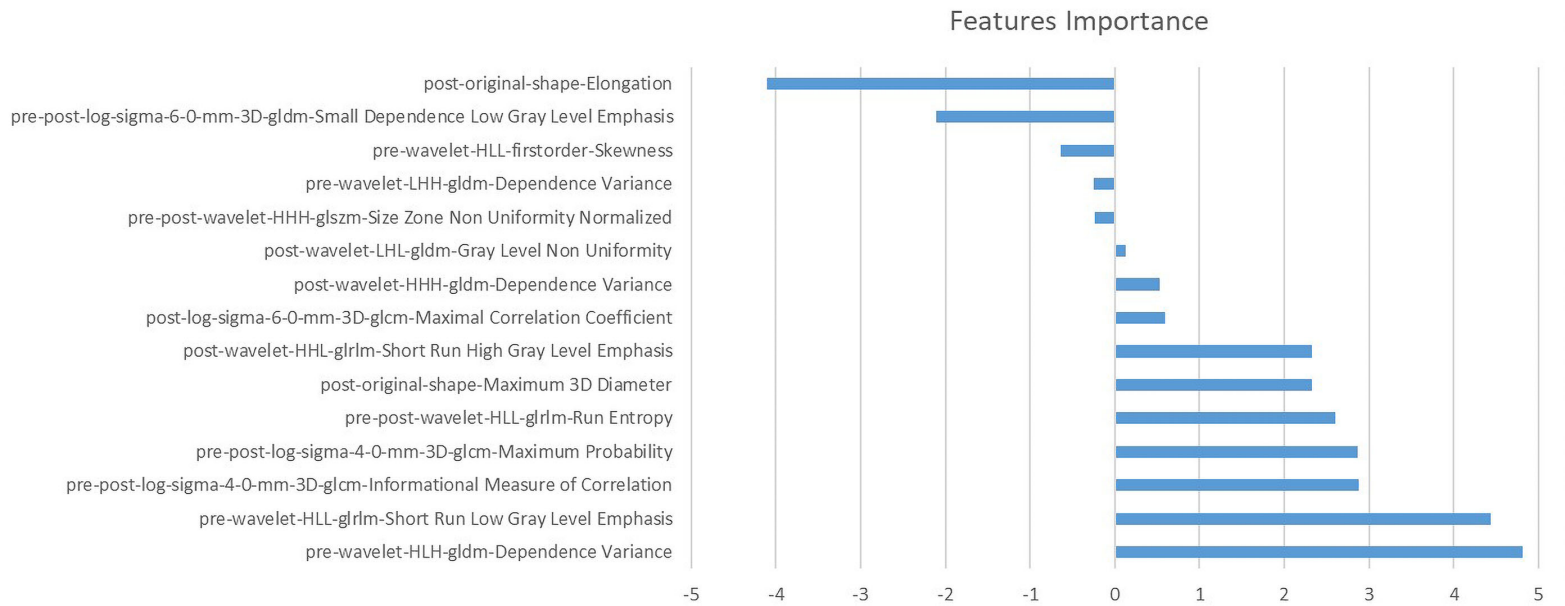
The importance of selected features by combination model.

The features of the combination model and their correlation coefficients are presented in **Table 5** and **Figure 3**. Among the 15 features of the combination model, 9/15 (60.0%) were wavelet-based features, 4/15 (26.7%) were LoG features, and 2/15 (13.3%) were shape features from the original images. Wavelet-based features generally have higher correlation coefficients than LoG features do. The four wavelet-based features with the highest correlation coefficients had higher correlation coefficients than the four LoG features with the highest correlation coefficients. Pre-NAC features accounted for 4/15 (26.7%) patients, post-NAC features accounted for 6/15 (40.0%), and pre-post-NAC features accounted for 5/15 (33.3%). Two of the four pre-NAC features had correlation coefficients greater than 2, accounting for 2/4 (50.0%); three of the six post-NAC features had correlation coefficients greater than 2, accounting for 3/6 (50.0%); and four of the five pre-post-NAC features had correlation coefficients greater than 2, accounting for 4/5 (80.0%). Pre-post-NAC features play a more important role in the combination model than pre-NAC and post-NAC features.

**Table 5:** Description of the selected radiomics features in combination model.

Radiomics feature	Radiomics group	Feature class filter	Image
Dependence Variance	gldm	wavelet-HLH	pre
Short Run Low Gray Level Emphasis	glrlm	wavelet-HLL	pre
Elongation	shape	original	post
Informational Measure of Correlation	glcm	log-sigma-4-0-mm-3D	pre-post
Maximum Probability	glcm	log-sigma-4-0-mm-3D	pre-post
Run Entropy	glrlm	wavelet-HLL	pre-post
Maximum 3D Diameter	shape	original	post
Short Run High Gray Level Emphasis	glrlm	wavelet-HHL	post
Small Dependence Low Gray Level Emphasis	gldm	log-sigma-6-0-mm-3D	pre-post
Skewness	first-order	wavelet-HLL	pre
Maximal Correlation Coefficient	glcm	log-sigma-6-0-mm-3D	post
Dependence Variance	gldm	wavelet-HHH	post
Dependence Variance	gldm	wavelet-LHH	pre
Size Zone Non Uniformity Normalized	glszm	wavelet-HHH	pre-post
Gray Level Non Uniformity	gldm	wavelet-LHL	post

Gldm, gray level dependence matrix; glrlm, gray level run length matrix; glcm, gray level co-occurrence matrix; glszm, gray level size zone matrix.

The linear combinations of combination features were as follows: RadScore = −2.757

+pre-wavelet-HLH-gldm-Dependence Variance × 4.812
+pre-wavelet-HLL-glrlm-Short Run Low Gray Level Emphasis × 4.436
-post-original-shape-Elongation × 4.101
+pre-post-log-sigma-4-0-mm-3D-glcm-Informational Measure of Correlation × 2.874
+pre-post-log-sigma-4-0-mm-3D-glcm-Maximum Probability × 2.87
+pre-post-wavelet-HLL-glrlm-Run Entropy × 2.607
+post-original-shape-Maximum 3D Diameter × 2.331
+post-wavelet-HHL-glrlm-Short Run High Gray Level Emphasis × 2.321
-pre-post-log-sigma-6-0-mm-3D-gldm-Small Dependence Low Gray Level Emphasis × 2.113
-pre-wavelet-HLL-firstorder-Skewness × 0.636
+post-log-sigma-6-0-mm-3D-glcm-Maximal Correlation Coefficient × 0.593
+post-wavelet-HHH-gldm-Dependence Variance × 0.531
-pre-wavelet-LHH-gldm-Dependence Variance × 0.253
-pre-post-wavelet-HHH-glszm-Size Zone Non Uniformity Normalized × 0.242
+post-wavelet-LHL-gldm-Gray Level Non Uniformity × 0.122.

## 4 Discussion

The pattern of tumor shrinkage is critical for determining which patients should be treated with BCS. We developed a combination model based on pre- and post-NAC DCE-MRI to predict the pattern of tumor shrinkage in our cohort of patients. This model can help clinicians to select suitable candidates for BCS. The performance of the combination model was superior to those of the pre-NAC, post-NAC, and pre-post-NAC models. High-order features contribute significantly to the radiomics model.

Previous studies have demonstrated that first-order features, shape features, and texture features can be used to predict tumor response to NAC (30, 31). Our proposed method extracts general radiomics features containing first-order, shape, and texture features (2D and 3D). Meanwhile, this method computes high-order features from filtered images with different filters. Sutton et al. (32) added Gabor features to the general radiomics features to predict pCR. However, Braman et al. (33) showed that only 2 of the 10 features used in the prediction models of hormone receptor+, HER2−, and TN/HER2+ were Gabor features with a lower correlation. In comparison, Gabor features were not highly correlated with the top 10 features in the all-comers prediction model (all subtypes were included). This discrepancy can be attributed to the inadequate processing capability of the Gabor filter for mutant and non-smooth signals.

The wavelet transform can compensate for the deficiency of the Gabor transform, which is a localized analysis of spatial frequencies. This fact can be applied to effectively extract high- and low-frequency signals from images and to analyze image texture changes more carefully and comprehensively. Zhou et al. (34) confirmed that wavelet-transformed textures can be used to predict pCR based on DCE-MRI. Thus, wavelet-based features were added to the models. Nine wavelet-based features were selected using the combination model.

Some scholars have suggested that LoG, which is designed to highlight the regions in an image where the intensity is changing rapidly, is compelling. Choudhery et al. (35) extracted 3D shape and texture features of TN breast cancer for analysis. They found that LoG features, including mean signal intensity, median signal intensity, maximum signal intensity, minimum signal intensity, and standard deviation of intensity, could be used to predict pCR. This study also included LoG features for analysis, namely the Informational Measure of Correlation, Maximum Probability, Small Dependence Low Gray Level Emphasis, and Maximal Correlation Coefficient. Our method uses both wavelet-based and LoG features to achieve better performance. These results suggest that high-order features have more potential for application in the prediction of BCS candidates.

In addition, among the features with correlation importance >2, the number of features from pre-post-NAC DCE-MRI surpassed pre- and post-NAC DCE-MRI. This showed that pre-post-NAC images can provide more effective information about the regression pattern of the tumor response to NAC. This is confirmed by the fact that the pre-post-NAC model is second only to the combination model in terms of predictive performance.

Most of the studies above were dedicated to predicting pCR and lacked further discussion of non-pCR tumors. The accurate identification of tumor remission patterns is critical for deciding the surgical approach. To accurately identify patients suitable for BCS, our study classified all tumor shrinkage patterns into types 1 and 2. Multifocal and major residual lesions with satellite foci are at risk of missing lesions during surgery, resulting in false-negative margins. Thus, they were combined with SD and PD, which do not respond well to NAC, to form type 2. The remaining pCR and single-lesion shrinkage were classified as type 1. The combination model used four LoG features, nine wavelet-based features, and two original shape features to classify shrinkage patterns with an AUC of 0.82.

Several studies that used pre-NAC MRI and MRI before and after NAC had predictive effectiveness similar to ours. Cain et al. (36) predicted the pCR of TN/HER2 patients based on pre-NAC DCE-MRI, with an AUC of up to 0.71. Sutton et al. (32) combined pre- and post-NAC DCE-MRI to establish a recursive feature elimination random forest model with AUC of 0.80 and 0.78 for training set and test set. However, these studies only discriminated between pCR and non-pCR patients. In our study, a further distinction was made between non-pCR and types 1 and 2. This approach was also presented in studies by Zhuang et al. (23) and Huang et al. (37). However, there was no comparison between the pre- and post-treatment images in their study. To this end, in addition to the combination model, we built the pre-NAC, post-NAC, and pre-post-NAC models separately for comparison. Our study showed that the performance of pre-post-NAC images was better than that of pre-NAC and post-NAC images. This suggests that changes in tumors before and after NAC treatment have significant predictive power in revealing the patterns of tumor regression. The above mentioned in our research is a further extension of previous research.

Ki-67 is a tumor proliferation marker with the gene located on the long arm of chromosome 10 (10q25) (38). Previous studies have concluded that higher Ki-67 levels in breast cancer are correlated with a better response to NAC (39, 40). Our findings showed that the regression pattern of Ki-67 >20% was more inclined towards type 1. However, the use of Ki-67 as an independent predictor requires further investigation.

Our study had several limitations. First, we did not include tumor subtypes as clinical features in the prediction model. This was because the tumor subtypes were not statistically significant in this study. The reason for this the distribution imbalance caused by the different incidence of each tumor subtype. Second, our proposed method can only be a component of the decision-making of the surgical approach for patients who have received neoadjuvant therapy, but other factors such as age, clinical nodal status, and tumor grade must be taken into consideration. Third, this was a pilot study, conducted with a sample size of 75 patients, and for validation, it required a larger population. Finally, this retrospective study must be evaluated for reproducibility and efficacy in a prospective validation set before its clinical application.

## 5 Conclusion

We constructed a combination model based on pre- and post-NAC DCE-MRI, utilizing general radiomics features, wavelet-based features, and LoG features to precisely predict tumor shrinkage patterns before surgery in our cohort of patients. High-order features, particularly texture features based on the wavelet transform filter, are significant for the prediction model. Pre-post-NAC features offer a better predictive efficacy than pre- and post-NAC features. The model can help clinicians select suitable candidates for BCS to reduce the likelihood of residual tumors at surgical margins. Further expansion of the sample size and separate discussion by tumor subtype would help improve this model.

## Data availability statement

The raw data supporting the conclusions of this article will be made available by the authors with the consent of the author’s organization. Requests to access the datasets should be directed to ZC, chenzhigeng7@163.com.

## Ethics statement

The studies involving human participants were reviewed and approved by the Review Board of the Second Hospital of Dalian Medical University. Written informed consent for participation was not required for this study in accordance with the national legislation and the institutional requirements.

## Author contributions

ZC performed the experiments and finalized the manuscript. MH, JL, and XQ analyzed the data. FH assisted the study. ZC and XL conceived and designed the experiments. All authors listed have made a substantial, direct, and intellectual contribution to the work and approved it for publication.
